# A fuzzy decision support model for the evaluation and selection of healthcare projects in the framework of competition

**DOI:** 10.3389/fpubh.2023.1222125

**Published:** 2023-08-08

**Authors:** Beata Gavurova, Miroslav Kelemen, Volodymyr Polishchuk, Tawfik Mudarri, Volodymyr Smolanka

**Affiliations:** ^1^Department of Addictology, First Faculty of Medicine, Charles University and General Teaching Hospital in Prague, Prague, Czechia; ^2^Department of Flight Training, Faculty of Aeronautics, Technical University of Košice, Košice, Slovakia; ^3^Department of Software Systems, Faculty of Information Technology, Uzhhorod National University, Uzhhorod, Ukraine; ^4^Technical University of Košice, Faculty of Mining, Ecology, Process Control and Geotechnologies, Košice, Slovakia; ^5^Department of Neurology, Neurosurgery and Psychiatry, Faculty of Medicine, Uzhhorod National University, Uzhhorod, Ukraine

**Keywords:** healthy cities, projects, expert evaluation, fuzzy sets, European Green Deal, industry 5.0, decision-making

## Abstract

Our research aims to support decision-making regarding the financing of healthcare projects by structural funds with policies targeting reduction of the development gap among different regions and countries of the European Union as well as the achievement of economic and social cohesion. A fuzzy decision support model for the evaluation and selection of healthcare projects should rank the project applications for the selected region, accounting for the investor's wishes in the form of a regional coefficient in order to reduce the development gap between regions. On the one hand, our proposed model evaluates project applications based on selected criteria, which may be structured, weakly structured, or unstructured. On the other hand, it also incorporates information on the level of healthcare development in the region. The obtained ranking increases the degree of validity of the decision regarding the selection of projects for financing by investors, considering the level of development of the region where the project will be implemented. At the expense of European Union (EU) structural funds, a village, city, region, or state can receive funds for modernization and development of the healthcare sector and all related processes. To minimize risks, it is necessary to implement adequate support systems for decision-making in the assessment of project applications, as well as regional policy in the region where the project will be implemented. The primary goal of this study was to develop a complex fuzzy decision support model for the evaluation and selection of projects in the field of healthcare with the aim of reducing the development gap between regions. Based on the above description, we formed the following scientific hypothesis for this research: if the project selected for financing can successfully achieve its stated goals and increase the level of development of its region, it should be evaluated positively. This evaluation can be obtained using a complex fuzzy model constructed to account for the region's level of development in terms of the availability and quality of healthcare services in the region where the project will be implemented.

## 1. Introduction

Although many statistics and numerous reports have declared an improvement in the average health level in the member countries of the European Union (EU) over the last several decades, health differences among segments of the population continue to persist in various parts of the EU, as well as differences between the most and the least advantaged regions (1, 2). In some cases, these differences are even more pronounced. Major health differences among various regions and between rural and urban areas have been confirmed to be present. Across the EU member countries, we observe a relationship between socioeconomic status and health of the population: that is, people with lower levels of education, lower-status job titles, or lower incomes tend to die younger and to have more health problems (3, 4). Gender also plays a significant role: although women live longer than men, they live more years in poorer health (5, 6). Everyday living conditions significantly affect health equality, as do the factors of technological development and spatial planning of the urban environment. In order to achieve health equity, it is fundamentally important to provide communities in urban and local areas with access to fundamental goods and to support the physical and mental health of the population while protecting the environment (7).

Schemes that focus on eliminating health inequalities can take several forms, and can be aimed either at disadvantaged sectors of the population or at reducing health differences across the entire population (8). Local public policies aiming to eliminate health inequalities can support the improvement of the physical and socioeconomic environment in disadvantaged areas in several ways: for instance, improving access to nutritious food, or enhancing housing quality, employment, physical activity, and so on Salmi et al. (9). Interventions aimed at improving the overall health status of the population may not affect the entire population. Therefore, the universal approaches applied in regional health policies must be supplemented with specific approaches that take regional specificities into consideration (10). It is important to constantly analyze the best practices in the implementation of effective interventions aimed at eliminating health inequalities, to look for new challenges for local interventions, and to share experiences with city and local government administrators (11, 12). These measures will aid in difficult decision-making processes and in effective national and regional policy implementation (13).

A recent study by The Lancet Public Health^1^ reports the contributions by critical factors that play a role in generating health inequalities as follows: a 35% share of the contributions can be attributed to income security and social protections; 29% to living conditions; 19% to a lack of education and low self-confidence; 10% to access to healthcare; and 7% to employment and working conditions. The heterogeneity of these factors clearly indicates the need to create policies that can influence each of these factors in order to support the elimination of health inequalities at various regional levels. Policies aiming to reduce health inequalities between countries will not be successful if they lead to the persistence of health disparities within countries, or to such disparities becoming even more pronounced.

Several financial mechanisms providing resources for the creation and implementation of effective tools to eliminate health inequalities have been created to solve these problems. Financial mechanisms for the distribution of EU resources are among these, and policymakers and public health administrators must therefore tackle difficult decision-making problems in the selection of appropriate health projects. Considering the multi-sector importance of the issue and its systemic and procedural interconnectedness, the decision-making processes aiming to select the most effective projects are highly demanding (14, 15). Many factors that have come to prominence may also vary based on demographic effects, population structure, economic development of the relevant region, the health profile of the relevant region, and so on. Therefore, it is necessary to develop and seek out the appropriate tools to support decision-making mechanisms that will help policymakers in healthcare and other domains to improve the corresponding decision-making processes (16, 17). These consistently relevant issues formed the motivation behind our research.

During the development of intelligent systems, subject knowledge is neither complete nor reliable. In addition, our research uses knowledge obtained from experts, which is subjective and vague in nature. However, the use of precise statistical methods does not account for verbal inaccuracies and subjective factors arising from the incorporation of expert-derived information. This problem, in turn, leads to inadequate representation of the knowledge base according to which further decisions are made. In the domain of artificial intelligence, problems dealing with fuzziness are solved using descriptions of fuzzy concepts by linguistic variables and corresponding membership functions. This enables data mining and decision-making based on knowledge gained from experts. The problem of multi-criteria evaluation of objects falls within the domain of selection problems, which are an integral subset of the problems involved in decision support systems. Thus, the formulation of a multi-criteria evaluation problem based on the theory of fuzzy sets is necessary to develop a decision support system.

Our research aims to support decision-making regarding the financing of healthcare projects by structural funds with policy goals involving reduction of the development gap among different regions and countries of the EU and the achievement of economic and social cohesion. A fuzzy decision support model for the evaluation and selection of healthcare projects should rank the project applications made by the selected region, accounting for the investor's wishes regarding the regional coefficient, in order to reduce the development gaps between regions. On the one hand, such a model should evaluate project applications based on specified criteria, which may be structured, weakly structured, or unstructured. On the other hand, it should also incorporate information on the level of healthcare development in the corresponding region. The obtained ranking would increase the validity of decisions regarding the selection of projects for financing by investors, taking into consideration the level of development of the region where the project will be implemented.

At the expense of the EU structural funds, a village, city, region, or state can receive funds for modernization and for development of the healthcare sector and all related processes. However, the financing of the implementation of such projects is a risky business. To minimize risks, it is necessary to put in place adequate support systems for decision-making in the assessment of project applications, as well as accounting for regional policy where the project will be implemented. Experience indicates that, based on the quality of project applications, applications from more developed regions or from capitals are more likely to receive funding. There are situations in which a project application from a less developed region may receive fewer points, even though it could have been successfully implemented within the available financial constraints. Under such circumstances, a less developed region can be prevented from reducing the development gap relative to more developed regions.

The primary goal of this study was to develop a complex fuzzy decision support model for the evaluation and selection of projects in the field of healthcare in order to reduce the development gap between regions.

Based on the above outline, we can form a scientific hypothesis for this research as follows. If the project selected for financing can successfully achieve its stated goals and increase the level of development of its region, a positive project evaluation can be claimed. This evaluation can be obtained using a complex fuzzy model that is constructed to account for the level of development of the region where the project is to be implemented, in terms of the availability and quality of healthcare services in that region.

## 2. Literature review

In recent years, many studies have tackled the issue of health inequalities and sought optimal solutions for the elimination of such inequalities. Mackenbach and Kunst (18) point out three main problems with these studies: first, the small number of countries included, which reduces the possibility of obtaining a global understanding of the problems from the perspective of particular country; second, the un-harmonized nature of the data collected from participating countries; and third, the fragmentation associated with the unavailability of intermediate data or evidence (19–22). Knowledge-sharing among countries has proven to be a necessary component in the process of the development of the EU member countries (23–25). Therefore, studies investigating this issue can be considered highly valuable, in that they highlight the status of significant societal problems and possibilities for solving them (26–28).

The outcomes of these studies, presented in the subsection “Importance of regional policies for eliminating health inequalities in various regions,” point to the importance of examining factors that affect decision-making mechanisms at the regional level and the importance of interdepartmental collaboration in the process of the creation and implementation of active policies. In the subsection “Fuzzy approaches as an optimal tool in project evaluation processes,” an overview is provided of studies that have examined the effects of applying fuzzy approaches in decision-making mechanisms for the selection of optimal projects. These approaches enable the solution of regional development problems, such as the elimination of health inequalities between regions or countries. Although these studies are quite heterogeneous in their content, they made it possible to clarify the state of the problem at the international level, thereby emphasizing the strong significance of the research topic discussed.

### 2.1. Importance of regional policies for the elimination of health inequalities between regions

Mackenbach and Kunst (29) have pointed out the extent of health inequalities related to the socioeconomic environment. They propose improving educational opportunities, income distribution, health literacy, and access to health care as the main tools to solve these inequalities. According to Helgesen et al. (4), examining the prerequisites and capacities of municipalities for the implementation of appropriate policies and measures is very important for reducing inequalities in health care. The importance of examining socioeconomic factors to support public health within municipalities has also been justified by Hagen et al. (30). The authors observed great potential for the reduction of health disparities between the regions through intermunicipal collaboration related to local health promotion, as well as through the creation of intersectoral working groups.

The importance of intersectoral collaboration in the elimination of health inequalities between regions has also been evaluated by Storm et al. (7). This kind of collaboration between the public health sector and social and physical policy sectors is essential for reducing regional health inequalities, but implementing it in local practice is relatively difficult. When the effects of health strategies implemented at the national and regional levels were examined, some countries, such as France, Portugal, Poland, and Germany, obtained highly positive results. However, the effects varied greatly in countries such as Spain, Italy, and Belgium (8), which also confirms the weakness of the governance system in a majority of the countries regarding the impact of mechanisms for reducing health inequalities and the problematic integration of health strategies between the national and regional levels.

Morrison et al. (10) have confirmed that socioeconomic inequalities in the domain of health in urban areas are large. According to the authors, local governments have several possibilities for the creation of adequate policies to reduce them. The perceptions of public policymakers and their beliefs about the implementation of urban public policies are important.

Guldbrandsson and Bremberg (11) have also described the problems with intersectoral collaboration in public health and with reducing health disparities in their study. The authors considered these intersectoral collaborations to be insufficient and recommended the coordination of these activities between the Ministry of Health and other ministries. Borrell and Vaughan (31) investigated the need for a combination of political will, technical capacities, and efforts by citizens in order to achieve success in policies aimed at reducing social disparities in health. Health inequalities can be addressed by appropriate health and social policies involving various community groups and the government.

Szymborski and Zatoński (32) have highlighted the need to investigate the universal and selective approach adopted by interventions aiming to eliminate health inequalities. According to the authors, health literacy also plays an important role in this process (33–35).

Finally, Diez et al. (13) reported in their study on the effects of regional interventions on health inequalities within European cities and observed that few local-level interventions address the socioeconomic determinants of health inequalities. The dominant determinants included in the interventions are healthcare, employment, and education. When examining the effectiveness of interventions, it is important to focus on the evidence base, participation, and intersectorality. Administrators reported perceiving the lack of funds and the sustainability of projects as the main obstacles. The authors appeal for strengthening of the capacities of administrators and for political leadership in the field of health management.

### 2.2. Fuzzy approaches as an optimal tool in the project evaluation processes

Mardani et al. (14) systematically investigated conventional and fuzzy decision-making techniques that are applied in solving health and medical problems. The authors found that the most frequently implemented decision-making techniques in healthcare were analytic hierarchy process (AHP) techniques and hybrid approaches. These techniques were primarily used to assess the quality of services in healthcare and the medical industry (36). Shaygan and Testik (37) employed fuzzy approaches to select projects aimed at eliminating the causes of underperformance. They considered the fuzzy analytical hierarchy process (FAHP) for decision-making, which is integrated with cause and effect diagrams, to be the optimal method.

Chatterjee et al. (38) drew attention to the risks involved in the construction of strategies based on project assortment and prioritization. Selection of the optimal project portfolio is a risky activity due to the lack of funds and due to nominal technology with the non-legal judgment of experts. The authors considered the use of an analytical hierarchy process in a fuzzy environment to be optimal for selection of the best projects, as it takes into account the multiple levels of project risk and a set of criteria and subcriteria.

Furthermore, Fouladgar et al. (39) used the FAHP and VIKOR techniques as optimal methods to calculate importance weights for evaluation criteria, and thus to rank a set of feasible projects. Bolat et al. (40) drew attention to the fact that the combination of FAHP and fuzzy multi-objective linear programming (FMOLP) is a suitable tool for supporting project selection. The complex model proposed by the authors takes into account the conflicting ideas of decision-makers about quantitative and qualitative criteria and evaluates projects in an integrated way. Rȩbiasz et al. (41) prefer a two-step evaluation model that combines fuzzy AHP and fuzzy TOPSIS for project evaluation. The authors established the advantage of this model through a case study.

Enea and Piazza (42) identified several limitations of the fuzzy AHP approach. Knowledge of these limitations when creating models will enable achievement of the best possible results in terms of certainty and reliability. Tulasi and Rao (43) positively evaluated several aspects of fuzzy AHP, primarily the fact that fuzzy AHP effectively examines data fuzziness. Mahmoodzadeh et al. (44) have proposed a new method for solving project selection problems through fuzzy AHP and implementation of the TOPSIS algorithm. Fuzzy AHP techniques significantly eliminate uncertainty in project evaluation, unlike the traditional AHP method.

Additionally, Mohammed et al. (45), based on the results of analyses, confirmed that the application of fuzzy AHP approaches will make it possible to obtain more accurate, scientific, and objective results in the evaluation of projects and will support the improvement of the quality level of project management. The authors also acknowledged the advantages of fuzzy AHP.

Tüysüz and Kahraman (46) evaluated information technology projects and stated that project risks are multidimensional, so they must be assessed through multi-attribute decision-making methods. Mohagheghi and Mousavi (47) state that fuzzy models are an optimal tool even in the evaluation of high-tech projects that are associated with a high level of risks and technological knowledge. The authors present a new decision-making model that operates under Pythagorean fuzzy set (PFS) uncertainty, applying their method to a real case study. This model employs last aggregation and avoids defuzzification until the final step of the process.

Oh et al. (48) propose a decision-making framework based on a fuzzy expert system that uses three tools: a strategic bucket for strategic resource allocation, scoring models for evaluating projects, and portfolio matrices for identifying the optimal set of projects in a portfolio. The final selection of projects was carried out by an expert system.

Jafarzadeh et al. (49) state that many methods have trouble accounting for three important criteria: selection criteria preference, decision uncertainty, and interdependencies. The authors propose a project evaluation method based on a combination of quality function development (QFD), fuzzy logic, and data envelopment analysis (DEA) to account for prioritization, uncertainty, and interdependence.

The selection of projects in the field of research and development is also a highly complex decision-making problem, as pointed out in a study by Mohanty et al. (50). The authors evaluated the importance of the opportunity environment, the impact of stakeholders on the evaluation, and the capacity of the candidate projects. In this area, important barriers also include bureaucratic factors, the different perceptions of the institution's goals by the pluralistic set of interested parties, and the functional specialization of organization members. These factors are significant obstacles to the achievement of coordination and consensus. The authors recommended applying fuzzy ANP (analytic network process) along with fuzzy cost analysis in the selection of research and development projects.

Bellahcene et al. (51) tackled with the selection of information systems projects, proposing an integrated AHP and a weighted-additive fuzzy goal programming (WAFGP) method as the optimal tools for their evaluation and selection. Similarly, Riddell and Wallace (52) tackled the problem of selecting a portfolio of research and development projects. The authors proposed a new tool to facilitate decision-making processes, which integrates fuzzy logic and expert judgment into the individual decision-making criteria for the decision-making process on projects in the research and development field. These authors also presented a real case study for illustration.

Mohagheghi et al. (53) evaluated studies focused on project portfolio selection and optimization, with an emphasis on evaluation criteria, applied approaches, uncertainty modeling, and application processes. The authors criticized the insufficient attention that has been paid to the issue of the development of decision-making methods in the area of projects in previous periods and the insufficient critical evaluation of available studies. According to the authors, expert systems, artificial intelligence, and big data science have not been given sufficient consideration or sufficiently widely applied in project selection processes.

Wu et al. (54) propose a project selection method based on stochastic dominance and fuzzy theory. The authors attempted to eliminate the subjective influences in risk assessment when estimating the expected value of the project portfolio.

The outcomes of these research studies clearly indicate the strong benefits of applying fuzzy methods or fuzzy techniques in the process of evaluating projects in various social areas. The multidisciplinary nature and strong systemic interconnectedness of health inequalities also create various dimensions of uncertainty, indeterminacy, and risks. Fuzzy approaches play a crucial role in addressing these challenges and supporting the achievement of optimal results in the decision-making processes of policymakers, strategic planning, and development plans. Review of these studies also revealed a clear research gap. Specifically, there is a significant lack of studies reporting on the use of fuzzy approaches in decision-making processes in the evaluation and selection of projects aiming to improve health across the regions and eliminate health inequalities at regional and national levels.

## 3. Materials and methods

### 3.1. Formal formulation of the evaluation problem

Let us define the following: *P* = {*P*_1_; *P*_2_; …; *P*_*n*_}, a set of projects in the healthcare field of a certain region for evaluation and selection for financing by investors; *C*_*P*_, an information model of the criteria for evaluating projects or scientific developments in the healthcare field; *C*_*R*_, an information model of evaluation criteria for the regions where projects will be implemented; and *M*_*P*_, a complex fuzzy mathematical model for evaluating projects in the healthcare field, considering the region of project implementation.

With these definitions, the system-theoretical-multiple model of the problem of evaluating and selecting projects in the healthcare field that will contribute to reducing the gap in development between regions, is presented as follows:


(1) $$\begin{array}{l} \left\{P,C_{P},C_{R},M_{P}|Y\left(f\right)\right\}. \end{array}$$

As a result, we obtain output estimates of *Y*(*f*), on the basis of which decisions are made on the financing of projects in the healthcare field, considering the level of development of the region. *Y*(*f*) = { μ (*P*), (*R*), *KR*}, where μ(*P*) is the score of the project application in the healthcare field; (*R*) is the level of development of the region where the project will be implemented; and *KR* is the “desire for a regional coefficient” on the part of investors, which allows for the support of projects in less developed regions, thereby reducing the gap in development among regions.

The following administrative agents are introduced for the given task: experts are individuals who analyze and evaluate the project applications; a project analyst is a person who configures and manages the evaluation process according to investors' needs, forms a set of evaluation criteria for relevant types of project in the healthcare field, and builds an information model of the evaluation criteria for project implementation regions; and investors are the managers of the corresponding structural or investment funds, who make decisions on the selection and financing of the evaluated projects and introduce additional parameters for selection.

**Figure 1:**
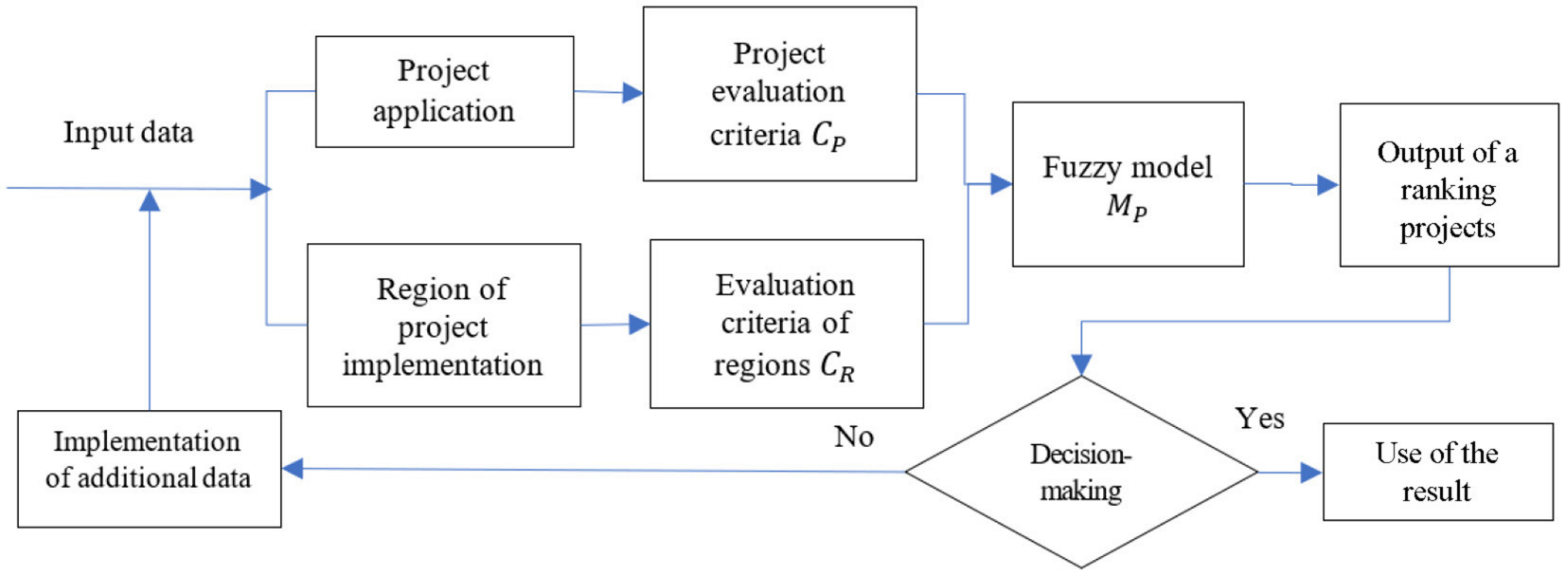
Structural scheme of the complex fuzzy model.

The structural scheme of the complex fuzzy model for evaluating projects in the healthcare field, accounting for the region of project implementation, is shown in Figure 1.

Figure 1 illustrates the structural scheme for the evaluation of projects in the healthcare field. After the evaluation and output of a ranking of projects, a decision is made regarding the selection. If the decision does not satisfy the investors, there is an opportunity to incorporate additional indicators and data for evaluation. At the output step, in order to increase the degree of reasonableness of decision-making regarding the investor's choice of projects for financing, a ranking of projects is provided, as well as their output evaluations, accounting for the region of project implementation and the policy of structured funds to reduce the development gap among regions.

### 3.2. Information models of project evaluation criteria and regions of project implementation

Information models based on which the submitted project applications will be evaluated are given below. Information models form the basis of the complex fuzzy model constructed to evaluate projects in the healthcare field, considering the region of project implementation.

*C*_*P*_–Information model of criteria for evaluating projects or scientific developments in the healthcare field.

This information model consists of four groups of criteria: *G* = {*G*_1_; *G*_2_; *G*_3_; *G*_4_}. Each group consists of its own set of criteria, and applications are scored on each criterion.

*G*_1_–*The idea and quality of the future project in the healthcare field*.

This group of criteria includes an assessment of the justification for the project, its focus on solving an actual problem in the social or scientific sphere of health care, the clarity of the formulation of the goal and tasks, and their compliance with the current level of innovative achievements.

*K*_11_–Relevance of the project to the development policies of the healthcare industry.

The relevance of the project, in terms of its correspondence to one or more cross-cutting priorities of the development of the healthcare sector, both at the state level and at the EU level, is substantiated.

*K*_12_–Relevance of the purpose, results, and target audiences.

The specified project description and its subsequent implementation will lead to expected products or services in the healthcare field, which will reflect its key priorities and attract expected target audiences.

*K*_13_–Motivation and validity of the project concept in the healthcare field.

The main focus is on whether the current state of research and the problems that need to be solved are described adequately and with appropriate references.

*K*_14_–Innovativeness of ideas (including from an interdisciplinary perspective).

*K*_15_–Adequacy of the proposed approaches and methods for project implementation and their compliance with the purpose and tasks of the project.

*G*_2_–*Significance of the project in the healthcare field for further development of the territorial community or society*.

Under this group of criteria, the rationale for the prospect of further application of the results obtained during the implementation of the project, or the possibility of commercialization of the project assets, is assessed.

*K*_21_–The potential importance of the expected results and the acquisition of new knowledge, the development of new approaches and technologies, and/or their importance for solving real, practical scientific/technical/social problems.

*K*_22_–The effectiveness and appropriateness of planned ways of publicizing/using project results.

*G*_3_–*The quality and realism of the proposed project implementation plan*.

Here, the reasonableness of the work plan and the clarity of the intermediate goals, as well as their logical sequence, are evaluated. This includes evaluation of the clarity of the description of the planned tasks with the indication of specific results that can be verified; consistency of the complexity of the tasks with their time frames; compliance of the equipment and materials specified as necessary for implementing the project, the realization of its purpose and tasks; and the clarity of the description of equipment and materials and the adequacy of their price in the budget.

*K*_31_–The validity of the work plan, the compliance of the time frame with the complexity of the formulated stages and tasks, the clarity of intermediate goals, and their logical sequence.

*K*_32_–Correspondence of the material and technical base and equipment (available and planned) to the assigned tasks.

*K*_33_–Balance and reasonableness of the overall project budget.

*K*_34_–Availability and reasonableness of an assessment of possible risks and prediction of ways to prevent or resolve them.

*G*_4_–*Subjects involved in project implementation in the healthcare field*.

Here, the validity of the qualitative composition of the subjects involved in project implementation and their partners, and their degree of preparedness to successfully make decisions regarding the declared goals of the project, is evaluated.

*K*_41_–The presence of a project partner or the intention to involve one.

Involved and/or potential partners representing an industry other than healthcare.

*K*_42_–Project team.

The composition of the project team is balanced between implementation of project tasks and project management; it includes all key performers responsible for the implementation of project tasks.

*K*_43_–Experience in project activities.

The project applicant's previous experience and activities meet the requirements and match the declared areas of activity.

The groups of criteria listed and their sets of sub-criteria are open and non-exhaustive, and the developed model does not depend on their number. This means that the project analyst can always add other important indicators, depending on the specifics of the projects or the subject of the competition.

Next, *C*_*R*_ is considered; this is an information model of evaluation criteria for project implementation regions.

This information model must be developed in consideration of the regions where the projects will be implemented. An example of the criteria for evaluation of the development of regions is given for regions of Ukraine. The information is taken from open data provided by the Ministry of Development of Communities and Territories of Ukraine (1). The indicators for “Availability and quality of services in the field of health care” are as follows:

*RC*_1_–Total mortality rate per 1,000 people of the existing population (per mille).

*RC*_2_–Average life expectancy at birth (years).

*RC*_3_–Number of live births per 1,000 people of the existing population (per mille).

*RC*_4_–Planned capacity of outpatient polyclinic facilities per 10,000 people (visits per shift).

*RC*_5_–Number of patients diagnosed with active tuberculosis for the first time in their lives per 100,000 population (persons).

Assessments according to the above criteria are quantitative and are not normalized. Validation of the research is carried out using real data obtained from open reports by the Ministry of Development of Communities and Territories of Ukraine.

The information model presented here is not a benchmark for assessment of the level of healthcare in the region. Instead, it demonstrates the possibility of formulating estimates for particular regions based on real data and is also used to verify and demonstrate a fuzzy model. The developed mathematical model does not depend on the number of evaluation criteria.

### 3.3. A complex fuzzy model for evaluating projects in the healthcare field, accounting for the region of project implementation

The mathematical model of project evaluation in the healthcare field, accounting for the region of project implementation, is presented in three stages: fuzzy evaluation of the project applications in the healthcare field; derivation of the level of development of the project implementation region; and derivation of output estimates of projects in the healthcare field, considering the level of development of the region and decision-making by investors regarding financing.

*First stage: fuzzy evaluation of the project application in the healthcare field*

According to the given information model consisting of the *C*_*P*_ criteria, an expert assigns the appropriate score for each criterion, for example, from the interval (1, 10). Such a score can be determined by the expert through analysis of the project application, using their own experience and practical knowledge. A convolution of evaluations can be determined, for example, as the sum of the scores for of the answers of the grading scale separately for each group of criteria; this can be denoted as *g*_*i*_, *i* = 1, *k*.

Thus, a set of numerical variables *g* = {*g*_1_; *g*_2_; …; *g*_*k*_} can be obtained for the group of evaluation criteria *G* = {*G*_1_; *G*_2_; …; *G*_*k*_}, taking values within a certain numerical interval. Each of these numerical variables is considered to be a carrier set of the linguistic variable *U*, consisting of the following terms:

- *U*_*i*1_–the evaluation of the group of criteria *G*_*i*_ is significantly lower than “investors' wishes”;
- *U*_*i*2_–the evaluation of the group of criteria *G*_*i*_ is lower than “investors' wishes”;
- *U*_*i*3_–the evaluation of the group of criteria *G*_*i*_ is close to “investors' wishes”;
- *U*_*i*4_–the evaluation of the group of criteria *G*_*i*_ is a little better than “investors' wishes”;
- *U*_*i*5_–the evaluation of the group of criteria *G*_*i*_ is much better than “investors' wishes.”

“Investors' wishes” is a conditional convolution of scores of a group of criteria that satisfies the investors when considering, evaluating, and choosing a project for financing.

Since the input data are obtained by expert means and are subjective by nature, it is necessary reveal uncertainty in the input data for the groups of criteria. This operation is referred to as fuzzification of input data. Next, we project the set of “investors' wishes” onto the carrier set of linguistic variables *U*.

Since the obtained numerical variables {*g*_1_; *g*_2_; …; *g*_*k*_} take different numerical values, it is necessary to calculate normalized values for comparison. To perform fuzzification of the input data, we construct a membership function of the type “Value x is greater”. For example, an s-shaped membership function will have the following form:


(2) $$\begin{array}{l} \mu (g_{ij})=\{0,\;g_{ij}\leq min;\;2{\left(\frac{g_{ij}-min}{max-min}\right)}^{2},\\ \;min<g_{ij}\leq \frac{min+max}{2};\;1-2{\left(\frac{max-g_{ij}}{max-min}\right)}^{2},\\ \;\frac{min+max}{2}<g_{ij}<max;\;1,\;g_{ij}\geq max. \end{array}$$

Here, *min* is the convolution of the sum of the minimum points (grades) and *max* is the convolution of the sum of the maximum points (grades) according to the criteria. In the group *G*_*i*_, *g*_*ij*_ is the convolution of the sum of the points (grades) for the *j-th* project under consideration(*i* = 1, *k*; *j* = 1, *n*). In this way, the received input data can be normalized and rendered comparable.

Let γ_*ij*_ = μ(*g*_*ij*_) be the value of the function of membership of the corresponding project applications by groups of criteria *G*_*i*_,(*i* = 1, *k*).

Let the investor have a set of considerations for each group of criteria, which should be regarded as the “investors' wishes”; that is, the sum of the points for each group of criteria. We denote these by the vector *T* = (*t*_1_, *t*_2_, …, *t*_*k*_) by groups of criteria *G*_*i*_, (*i* = 1, *k*). Similarly, for each value, the membership function can be calculated according to formula (2). Consequently, the vector of the membership function “investors' wishes” can be obtained as α = (α_1_, α_2_, …, α_*k*_), where α_*i*_ = μ(*t*_*i*_), (*i* = 1, *k*). The obtained values are given in Table 1.

**Table 1:** Fuzzification of input data.

**Groups of criteria**	**“Investors' wishes”**	***P*_1_**	***P*_2_**	**…**	***P*_*n*_**
*G*_1_	(*t*_1_; α_1_)	(*g*_11_; γ_11_)	(*g*_12_; γ_12_)	…	(*g*_1*n*_; γ_1*n*_)
*G*_2_	(*t*_2_; α_2_)	(*g*_21_; γ_21_)	(*g*_22_; γ_22_)	…	(*g*_2*n*_; γ_2*n*_)
…	…	…	…	…	…
*G*_*k*_	(*t*_*k*_; α_*k*_)	(*g*_*k*1_; γ_*k*1_)	(*g*_*k*2_; γ_*k*2_)	…	(*g*_*kn*_; γ_*kn*_)

Next, relative to the “investors' wishes” and the results obtained for each group of criteria *G*_*i*_, the values of the membership function are projected onto the set of carriers of the linguistic variable *U*, which allows us to reveal the essence of the considered project application in relation to the “investors' wishes”. Therefore, for each term *U*, the construction of triangular membership functions can be proposed, as follows:


(3) $$\begin{array}{l} \mu_{U1}\left(\gamma ;\alpha -\frac{\alpha }{2};\alpha -\frac{\alpha }{4}\right)=\{1,\;\gamma \leq \alpha -\frac{\alpha }{2};\;\frac{3\alpha -4\gamma }{\alpha },\\ \;\alpha -\frac{\alpha }{2}<\gamma \leq \alpha -\frac{\alpha }{4}. \end{array}$$

(4) $$\begin{array}{l} \mu_{U2}\left(\gamma ;\alpha -\frac{\alpha }{2};\alpha -\frac{\alpha }{4};\alpha \right)=\{\frac{4\gamma -2\alpha }{\alpha },\;\alpha -\frac{\alpha }{2}<\gamma \leq \alpha -\frac{\alpha }{4};\\ \;\frac{4\alpha -4\gamma }{\alpha },\;\alpha -\frac{\alpha }{4}<\gamma \leq \alpha . \end{array}$$

(5) $$\begin{array}{l} \mu_{U3}\left(\gamma ;\alpha -\frac{\alpha }{4};\alpha ;\alpha +\frac{\alpha }{4}\right)=\{\frac{4\gamma -3\alpha }{\alpha },\;\alpha -\frac{\alpha }{4}<\gamma \leq \alpha ;\\ \;\frac{5\alpha -4\gamma }{\alpha },\;\alpha <\gamma \leq \alpha +\frac{\alpha }{4}. \end{array}$$

(6) $$\begin{array}{l} \mu_{U4}\left(\gamma ;\alpha ;\alpha +\frac{\alpha }{4};\alpha +\frac{\alpha }{2}\right)=\{\frac{4\gamma -4\alpha }{\alpha },\;\alpha <\gamma \leq \alpha +\frac{\alpha }{4};\\ \;\frac{6\alpha -4\gamma }{\alpha },\;\alpha +\frac{\alpha }{4}<\gamma \leq \alpha +\frac{\alpha }{2}. \end{array}$$

(7) $$\begin{array}{l} \mu_{U5}\left(\gamma ;\alpha +\frac{\alpha }{4};\alpha +\frac{\alpha }{2}\right)=\{\frac{4\gamma -5\alpha }{\alpha },\;\alpha +\frac{\alpha }{4}<\gamma \leq \alpha +\frac{\alpha }{2};\\ \;1,\;\gamma \geq \alpha +\frac{\alpha }{2}. \end{array}$$

Depending on which interval γ falls into for each group of criteria *G*_*i*_, the appropriate membership function μ_*U*_ is selected relative to the “investors' wishes” of α. As a result, for each group of criteria *G*_*i*_ for all projects *P*_*j*_, we obtain a linguistic value and its confidence assessment. The confidence assessment means that the assessment of the group of criteria belongs to one term or another. This allows the model to reveal the subjectivity of opinions regarding the assignment of points by experts and to construct a formal representation of the quality of the project application.

Since the constructed membership functions (3)–(7) have intersections, either one or two terms are obtained for each group of criteria and, accordingly, the same number of reliability estimates are obtained for them. In this regard, we offer the following aggregation function:


(8) $$\begin{array}{l} \mu (O_{ij})=\{\mu_{Uijf}\cdot \sigma_{ijf},\;if\;one\;term,\;\mu_{Uijf}\cdot \sigma_{ijf}\\ \;+\mu_{Uij\left(f\pm 1\right)}\cdot \sigma_{ij\left(f\pm 1\right)},\;if\;two\;terms,\; \end{array}$$

where *i* = 1, *k*; *j* = 1, *n*; *f* = 1, 5; and σ_*ijf*_ is determined by the characteristic function on the interval [1; 100]: for example,


(9) $$\begin{array}{l} \sigma_{ijf}=\{50\;if\;U_{ijf}=U_{ij1};\;75\;if\;U_{ijf}=U_{ij2};100\;if\;U_{ijf}=U_{ij3};\\ \;75\;if\;U_{ijf}=U_{ij4};\;50\;if\;U_{ijf}=U_{ij5}.\; \end{array}$$

Without reducing generality, the project analyst can choose a different possible interval, as well as an alternative approach for the transition from linguistic to quantitative evaluation. The construction of such a characteristic function stems from the authors' own experience, as well as from the consideration that, as a rule, the final evaluation of project applications is provide on a 100-point scale. The output of the characteristic function decreases when the evaluation of the groups of criteria is further away from the “investors' wishes.”

The obtained membership function μ(*O*_*ij*_) shows to what extent the project application under consideration satisfies the wishes of the investors according to each group of criteria.

The investor may need to set the weighting coefficients {*p*_1_, *p*_2_*, …, p*_*k*_} for each group of criteria on the interval (1, 10). If there is no such need, then the weighting coefficients will be considered balanced. Next, normalized weighting factors for each group of criteria are determined:


(10) $$w_{i}=\frac{p_{i}}{\sum_{i=1}^{k}p_{i}},\;i=\underline{1,k};w_{i}\in \left[0,1\right]$$

meeting the condition $\sum_{i=1}^{k}w_{i}=1$.

A weighted average convolution is proposed to obtain the output estimate for the healthcare project application μ(*P*):


(11) $$\mu (P_{j})=\sum_{i=1}^{k}w_{i}\cdot \mu (O_{ij}),i=\underline{1,\;k};j=\underline{1,\;n}.$$

Thus, for all projects under consideration, we obtain an output estimate for the project application within the interval [0; 100].

*Second stage: determination of the level of development of the region where the project will be implemented*

As mentioned above, our research is aimed at supporting decision-making regarding the financing of healthcare projects by structural funds whose policy involves reducing the development gap among different regions. Therefore, a feature of the presented model is that it can adequately account for policies aimed at reducing the development gap among regions. To this end, the level of development of the region where the project will be implemented is derived from the mathematical model as (*R*). This is presented in Table 2, which represents input assessments of regions according to *C*_*R*_, the information model of the assessment criteria for project implementation regions.

**Table 2:** Table of evaluations of regions by criteria.

	***R*_1_**	***R*_2_**	**…**	***R*_*r*_**
*RC*_1_	*C*_11_	*C*_12_	…	*C*_1*r*_
*RC*_2_	*C*_21_	*C*_22_	…	*C*_2*r*_
…	…	…	…	…
*RC*_*rc*_	*C*_*rc*1_	*C*_*rc*2_	…	*C*_*rcr*_

Here, *C*_*rcr*_ is an estimate of the rc-th criterion for the r-th region, and is quantitative and not normalized. In all cases, *r* ≤ *n*, since several projects may be submitted for consideration from the same region. In general, this assessment can be made quantitatively on different assessment scales, or even qualitatively. In this case, it is necessary to standardize the data. To do so, we can use the method of displaying fuzzy knowledge that is described in Petrovic et al. (2). For the normalization of quantitative data, we use the relative normalization formula:


(12) $$O_{rcr}=\frac{C_{rcr}}{\max_{r}C_{rcr}}\;or\;O_{rcr}=\frac{\min_{r}C_{rcr}}{\;C_{rcr}}.$$

Let the investor or project analyst set the weighting coefficients for each regional criterion *{v*_1_, *v*_2_, …, *v*_*rc*_} on the interval [1, a]. Following this, the normalized weighting coefficients can be determined similarly for each criterion:


(13) $$\varepsilon_{g}=\frac{v_{g}}{\sum_{g=1}^{rc}v_{g}},g=\underline{1,rc};\varepsilon_{g}\in \left[0,1\right],$$

meeting the condition $\sum_{g=1}^{rc}\varepsilon_{g}=1$.

To deduce the level of development of the region, the following multiplication is proposed:


(14) $$\Delta_{j}(R_{h})=\sum_{g=1}^{rc}\alpha_{g}\cdot O_{hg},h=\underline{1,r},\;j=\underline{1,n}.$$

*Third stage: derivation of aggregated estimates for projects in the healthcare field, accounting for the level of development of the region and decision-making by investors on their financing*

Based on practical experience, more developed regions submit better project applications. This is due to many factors: for example, the region's adherence to modern trends, best practices, the experience of the project implementation team, access to a wide range of partners, experience in implementing similar projects, greater financial opportunities in co-financing, and so on. Therefore, to reduce the gap in the development of regions, it is proposed that investors specify a “desire for a regional coefficient”, i.e., *KR* ∈ [0; 1]. Specifically, if *KR* approaches 1, then investors are interested in the most developed regions, and vice versa. This will allow for rapid adjustment of the weight placed by investors on the regions where future projects are to be implemented. Regarding the “desire for a regional coefficient”, we calculate the following values, which will represent a relative estimate of the proximity of the “desire for a regional coefficient” to the value of the corresponding level of development of the region:


(15) $$\begin{array}{l} \chi (R_{h})=1-\frac{\left|KR-\Delta \left(R_{h}\right)\right|}{\left\{\;KR-\min_{h}\Delta \left(R_{h}\right);\max_{h}\Delta (R_{h})-KR\right\}},\\ \;h=\underline{1,r},\;j=\underline{1,n}. \end{array}$$

To derive the output estimates for projects in the healthcare field, accounting for the level of development of the region and the decision-making by investors on financing, we use the following formula:


(16) $$Y\left(f_{j}\right)=\;\mu \left(P_{j}\right)\cdot \chi_{j}\left(R\right),\;j=\underline{1,n}.$$

Based on the output score *Y* (*f*_*j*_) ∈ [0; 100], a ranking of projects *P*_*j*_ is constructed.

The obtained output estimate *Y*(*f*) incorporates the content of the project assessment, accounting for the policy of reducing the gap in the development of regions. Based on the output data, investors make decisions on the feasibility of financing healthcare projects, accounting for the level of development of the region where the project is to be implemented. If a situation arises in which the investors are not satisfied with any of the solutions, then we return to re-evaluation with the involvement of additional indicators and data.

The value of the model lies in enabling us to understand the essence of the evaluated project within the space of assessments, accounting for the wishes of the investors at various stages of assessment, including the region where the project will be implemented. The use of fuzzy set theory is another significant advantage, as it allows the subjectivity of expert determinations to be revealed, in order to obtain a quantitative assessment of projects based on fuzzy expert data inputs. Making a reasoned decision is possible only based on quantitative initial data. Another advantage is the ability to easily adjust the parameters of the model depending on the evaluation purpose and to enter the desired regional coefficient, which collectively reduces the subjective influence of project analysts or experts on the evaluation process and the final result of project selection by investors. Without reducing the generality, other researchers can use other methods of multi-criteria alternative selection, such as the hierarchy analysis method, fuzzy TOPSIS, or VIKOR. However, in order to incorporate the desired regional coefficient, special procedures must be introduced, entailing additional calculations. This will make the calculations involved in using the decision support system much more difficult, and in turn, increase the difficulty of implementing it in practice. In addition, with a large number of projects, for example, the hierarchy analysis method will not achieve adequate results. The disadvantages of this approach include the use of different types of membership functions, characteristic functions, and convolutions, which may lead to ambiguity in the final results. However, these disadvantages will not affect the reliability of the results.

## 4. Results

We verify and test the results of the research on the example of the evaluation of five projects in the field of healthcare, *P* = (*P*_1_; *P*_2_; …; *P*_5_), which are implemented at the expense of state funds of Ukraine (3, 4):

- *P*_1_–Personalized approaches to the diagnosis, prevention, and treatment of vascular diseases with prognostic modeling of the individual development of atherosclerosis.
- *P*_2_–Study of the course and consequences of COVID-19 in patients with diabetes and the impact of SARS-CoV-2 infection on the rate of biological aging.
- *P*_3_–Study of the circulation of zoonotic influenza A viruses in the natural reservoir and assessment of their epidemic risks and danger to human health in Ukraine.
- *P*_4_–Development of new anesthetic agents.
- *P*_5_–Drosophila melanogaster as a platform for screening new antiviral compounds and studying cellular mechanisms of defense against viruses.

Project *P*_1_ is currently being implemented at the expense of the state budget of Ukraine at the Uzhhorod National University (region: Zakarpattia) (3), and the remaining projects *P*_2_−*P*_5_ are being implemented at the expense of grant support from the National Research Fund of Ukraine (4).

The evaluation calculation is carried out based on the complex fuzzy model developed for evaluation of projects in the healthcare field, accounting for the region of project implementation. For this purpose, the assessment is carried out via the three stages described above. Such an evaluation was carried out by the authors of this article, who are experts in various commissions and competitions for the evaluation of grants, scientific, technical, and startup projects.

As an example, consider in more detail the evaluation of a project application for a project *P*_1_.

First stage: fuzzy evaluation of the project application in the healthcare field.

At the first stage of assessment of project *P*_1_, we receive the input data for each evaluation criterion according to *C*_*P*_, the information model of evaluation criteria for projects or scientific developments in the healthcare field. *G*_1_ represents the idea and the quality of the project (*K*_11_ = 9; *K*_12_ = 10; *K*_13_ = 8; *K*_14_ = 7; *K*_15_ = 10; *g*_11_ = 44); *G*_2_ represents the significance of the project for further development of society (*K*_21_ = 10; *K*_22_ = 8; *g*_21_ = 18); *G*_3_ represents the quality and realism of the proposed project implementation plan (*K*_31_ = 9; *K*_32_ = 10; *K*_33_ = 10; *K*_34_= 7; *g*_31_ = 36); and *G*_4_ represents the subjects involved in project implementation (*K*_41_ = 8; *K*_42_ = 10; *K*_43_ = 10; *g*_41_ = 28).

Formula (2) is used for fuzzification of the input data. The following is thereby obtained for $G_{1}:min=5,\;max=50,\;\mu \left(g_{11}\right)=\;1-2{\left(\frac{50-44}{50-5}\right)}^{2}\approx 0.97.\;Similarly,\;\mu \left(g_{21}\right)=1-2{\left(\frac{20-18}{20-2}\right)}^{2}\approx 0.98;\;\mu \left(g_{31}\right)=1-2{\left(\frac{40-36}{40-4}\right)}^{2}\approx 0.98;\;\mu \left(g_{41}\right)=1-2{\left(\frac{30-28}{30-3}\right)}^{2}\approx 0.99.$

Next, for each group of criteria, investors express their “investors' wishes” according to four groups of criteria: *T* = (40; 18; 37; 25). Similarly, the membership function is calculated for each value according to formula (2): α = (0.9; 0.98; 0.99; 0.93).

Subsequently, the values of the membership function relative to the “investors' wishes” and the results obtained for each group of criteria are projected onto the set of carriers of the linguistic variable *U* according to formulas (3)–(7):

*G*_1_: *U*_113_ with confidence μ_*U*113_ = 0.69 or *U*_114_ with confidence μ_*U*114_ = 0.31.

*G*_2_: *U*_212_ with confidence μ_*U*212_ = 0 or *U*_213_ with confidence μ_*U*213_ = 1.

*G*_3_: *U*_312_ with confidence μ_*U*312_ = 0.04 or *U*_313_ with confidence μ_*U*313_ = 0.96.

*G*_4_: *U*_413_ with confidence μ_*U*413_ = 0.78 or *U*_414_ with confidence μ_*U*414_ = 0.22.

Next, the extent to which the project application under consideration satisfies the wishes of the investor according to each group of criteria is calculated using formula (8):

*μ*(*O*_11_) = 0.69 · 100 + 0.31 · 75 = 92.25; *μ*(*O*_21_) = 0 · 75 + 1 · 100 = 100; *μ*(*O*_31_) = 0.04 · 75 + 0.96 · 100 = 99; *μ*(*O*_41_) = 0.78 · 100 + 0.22 · 75 = 94.5.

Let the investor set the weighting coefficients for each group of criteria {10; 9; 8; 8}. On this basis, the normalized weighting factors calculated by formula (10) are: *w*_1_ = 0.28; *w*_2_ = 0.26; *w*_3_ = 0.23; *w*_4_ = 0.23.

Finally, a weighted average convolution according to the formula is used to obtain the output estimate for the project application (11): *μ*(*P*_1_) = 0.28 · 92.25 + 0.26 · 100 + 0.23 · 99 + 0.23 · 94.5 = 96.4.

Information on the evaluation of the remaining projects *P*_2_−*P*_5_ is provided in the official results of the competition (5): *μ*(*P*_2_) = 96.1; *μ*(*P*_3_) = 94.4; *μ*(*P*_4_) = 93.4; *μ*(*P*_5_) = 92.8.

*Second stage: determination of the level of development of the region where the project will be implemented*

The indicators of “availability and quality of services in the field of healthcare” (1) according to the above criteria for the regions corresponding to the projects under consideration are presented in Table 3. Information on indicators for Ukraine is also provided in the table, for purposes of normalization of the quantitative data, as well as the weighting factors for each regional criterion set by the project analyst.

**Table 3:** Input data on “availability and quality of services in the field of healthcare”.

**Criterion label**	**Weight**	**Values for Ukraine**	***P*_1_**	***P*_2_, *P*_4_**	***P*_3_**	***P*_5_**
			*R*_1_	*R*_2_	*R*_3_	*R*_4_
			**Zakarpattia**	**Kyiv**	**Kharkiv region**	**Lviv region**
*RC*_1_	10	14.8 (min)	14.8	15.3	21	16
*RC*_2_	9	73.5 (max)	70.47	73.5	71.11	72.42
*RC*_3_	9	10.2 (max)	10.1	10	5.9	7.8
*RC*_4_	8	306.8 (max)	250.6	287.9	298.1	201.6
*RC*_5_	7	18.4 (min)	44	24.5	28.2	35.3

In the next step, the relative normalization formula (12) is used to normalize quantitative data. Normalized weighting factors for each criterion are also determined according to formula (13). The results are shown in Table 4.

**Table 4:** Normalized data on “availability and quality of services in the field of healthcare”.

**Criterion label**	**Normalized data**	***P*_1_**	***P*_2_, *P*_4_**	***P*_3_**	***P*_5_**
		**R**_**1**_	**R**_**2**_	**R**_**3**_	**R**_**4**_
		**Zakarpattia**	**Kyiv**	**Kharkiv region**	**Lviv region**
*RC*_1_	0.23	1	0.967	0.705	0.925
*RC*_2_	0.21	0.959	1	0.967	0.985
*RC*_3_	0.21	0.99	0.98	0.578	0.765
*RC*_4_	0.19	0.817	0.938	0.972	0.657
*RC*_5_	0.16	0.418	0.751	0.652	0.521

To derive the level of development of the region, the membership functions are calculated using formula (14): Δ_1_(*R*_1_) = 0.861; Δ_2_(*R*_2_) = 0.936; Δ_3_(*R*_3_) = 0.774; Δ_4_(*R*_2_) = 0.936; Δ_5_(*R*_4_) = 0.789.

*Third stage: derivation of aggregated estimates for projects in the healthcare field, accounting for the level of development of the region and decision-making by investors on financing*

In pursuit of the goal of reducing gaps in development between the regions, the investors specify their “desire for a regional coefficient”. For example, let us set this coefficient at the value of *KR* = *0.8*. Using formula (15), values are calculated to provide a relative estimate of the proximity of the value of this “desire for a regional coefficient” to the corresponding level of development of the region: χ(*R*_1_) = 0.56; χ(*R*_2_) = 0; χ(*R*_3_) = 0.81; χ(*R*_4_) = 0.92.

The following formula is used to derive output estimates for the projects (16):


$$\begin{array}{l} Y\left(f_{1}\right)=96.4\cdot 0.56=53.59;\;Y\left(f_{2}\right)=0;\;Y\left(f_{3}\right)=76.71;\\ \;Y\left(f_{4}\right)=0;Y\left(f_{5}\right)=84.97. \end{array}$$

Finally, based on the obtained estimates, we can construct a ranking of projects, accounting for the policy of reducing the gaps in development between the regions: *P*_5_, *P*_3_, *P*_1_, *P*_2_, *P*_4_.

The investor concludes that, with the “desire for a regional coefficient” set to 0.8, the best decision in terms of the selection of a project for financing and implementation will be project *P*_5_.

## 5. Discussion

In the study, a fuzzy model of support for decision-making in the evaluation and selection of projects in the healthcare field was developed to reduce the development gap between regions. For this purpose, the following components were developed: an information model of criteria for evaluating investment projects or scientific developments in the healthcare field; a model of criteria for evaluating regions where projects are planned to be implemented in terms of the level of development of these regions; and a complex fuzzy mathematical model for evaluating projects in the healthcare field, with the region of project implementation taken into consideration. The resulting model was tested on an example evaluation of five projects in the healthcare field.

This research is based on the apparatus of fuzzy sets, which allows for increasing the degree of validity of decisions. The value of the model is that it allows the user to obtain a quantitative assessment of projects based on fuzzy input expert data, accounting for the wishes of the investor at different stages of the assessment, the policy of reducing development gaps among regions, and the wishes of the investor regarding the regional coefficient. The evaluation procedure itself is simple and natural for experts to implement. The input data are processed by a fuzzy model based on information models for evaluating project applications and regions. This reveals the subjectivity of experts' evaluations and establishes the model parameters and regional coefficients in order to reduce the subjective influence of project analysts or experts on the evaluation process and the final selection of projects by investors. The output of the model takes the form of a quantitative assessment and a ranking of the candidate healthcare projects for investors to choose from to pursue the goal of reducing the development gap between regions.

The advantages of a fuzzy decision support model for the evaluation and selection of projects in the healthcare field with the goal of reducing the development gap between regions arise from several features: (1) the mathematical model is based on various information models of input data adapted for the evaluation of projects or scientific developments in the healthcare field; (2) the sets and groups of criteria are open; (3) the model does not depend on their number, and project analysts can always adapt the set of criteria to highly specialized project topics; (4) the model makes it possible to understand the essence of the proposed project within the assessment space; (5) the model can easily be adjusted depending on the purpose of the assessment; (6) this approach reveals the uncertainty in the input data (expert estimates) using the carrier set of the linguistic variable *U* relative to “investors' wishes”; (7) it considers the wishes of the investor regarding the region where the project will be implemented by specifying a “desire for a regional coefficient”; and (8) it focuses on the unbiased assessment of projects, which increases the security of their financing overall.

It should be noted that the outputs of this fuzzy decision-making support model for the evaluation and selection healthcare projects are dependent on the meaning of the “investors' wishes”. This means that the maximum input values for a project do not mean a high output value. In addition, the input data undergo fuzzification, after which the values of the membership function are projected onto the set of carriers of the linguistic variable *U*. Through this procedure, errors of both external and internal origin can be avoided. Therefore, the decision support system is robust.

A limitation of our study is the use of different types of membership functions, characteristic functions, and convolutions, which may lead to ambiguity in the final results. Nevertheless, this limitation did not affect the reliability of the results obtained. This is supported by the research findings and the justified use of the fuzzy sets apparatus. The rationality of the obtained initial estimate *Y*(*f*) for building a ranking series of projects also demonstrates the advantages of the model developed. Furthermore, the results obtained fully conform to the research hypothesis formulated.

The development of tools to support decision-making techniques in the field of health represents a powerful mechanism in the process of reducing of health inequalities between regions (55). However, no tool can replace collaboration networks applying a multidisciplinary approach, alongside collaboration between sectors (56, 57). Many studies have claimed that urban administrators perceive health inequalities and understand the concepts of intersectorality, participation, and evidence, but considerate has become increasingly important to consider the sustainability of health systems at the regional level (58, 59).

Regional health policies require constant attention and are dependent on collaborative systems, data registries, and collaboration with various regional and national health associations (60–62). Diez et al. (13) recommended strengthening the capacities of administrators and of the regional political leadership in the healthcare field. If city governments want to make progress on policies aiming to reduce health inequalities, they need strong political commitment and support from social movements, including the support of public health experts. As stated by Borrell et al. (31), the policy agenda must include the goals in the area of health inequalities at different regional levels. Bekken et al. (63) drew attention to the insufficient capacity for effective activities aiming toward reducing health inequalities in smaller municipalities within defined regional units. Furthermore, they regarded a weak knowledge base as a critical factor, including the absence of systems for monitoring of social inequalities. New legislation in the domain of health may represent significant opportunities, and many countries are constantly developing such legislation in order to adapt to demographic trends and globalization risks. Morrison et al. (10) recommend supporting academic research on the creation of effective universal policies and evaluating their impact. This could also support the development of regional benchmarking indicators.

To reduce inequalities in the healthcare field, intersectoral collaboration will still be necessary (specifically, collaboration between the public health sector and other sectors), and this is often difficult to implement in practice. High-quality interdepartmental collaboration can also enable decision-making processes in the selection of effective interventions in the healthcare field while simultaneously eliminating resource and preference risks in the decision-making mechanisms. Therefore, it is necessary to investigate the potential for this collaboration, which already exists in the regions, because its absence can represent a significant barrier to the implementation of quality projects targeting the promotion of health. Storm et al. (7) also call for the involvement of these sectors in the network of public health policies, along with the harmonization of goals, the use of policies by relevant sectors, and formalized collaboration.

There is no optimal model for effective collaboration, so the success of health policies depends on the synergy between individual components of regional development systems. Many studies have claimed that space for improvement of this collaboration among sectors at regional and national levels will always be created, but it is necessary to seek out the most effective forms of collaboration in the context of the political goals being supported. This study creates a space for subsequent research in this area.

The fuzzy model for decision-making support developed by us for the evaluation and selection of projects in the healthcare field will help reduce the development gap in regions and ensure their sustainability. If projects are positively evaluated based on this comprehensive fuzzy model (which accounts for several aspects of the health system, including the availability and quality of healthcare provision), this is an indication that their outcomes can successfully fulfill the development goals set by health policy by and national and regional strategies. This will contribute to the reduction of regional and national disparities in population health, which will be reflected in macroeconomic indicators (64). From a macroeconomic perspective, the appropriate selection and successful implementation of ambitious projects in the healthcare field, which follow the resources and processes of the health sector, will help with the achievement of economic and social stability within and among countries. The developed model will be a useful tool for administrators and project analysts in such decision-making processes; it can be used to avoid ineffective financing of projects and to obtain resources from EU funds. From a long-term perspective, the need to develop tools that can be used to compare and evaluate the effectiveness of decision-making processes in various sectors when using resources drawing on EU funds has been highlighted, leading to the development of benchmarking indicators (65, 66). Their absence in the healthcare system to date is the result of not only the methodological complexity and strong heterogeneity of the healthcare systems, but also a lack of effort on the part of research teams to search for optimal decision-making mechanisms and indicators for their evaluation (67). Thus, the development of benchmarking indicators to enable the evaluation of the effects of implemented projects is an additional topic of research. Finally, these issues will also be related to an examination of their relationships with sustainable development goals (SDGs-17), which have not been sufficiently explored in existing studies. Inadequate fulfillment of the SDG-17 goals within individual countries has often been the subject of criticism among experts and in research. Financial schemes backed by EU funds can also help to solve these problems.

## 6. Conclusions

Sustaining good health and enhancing the quality of life of the population is the primary objective not only of the EU, but of all institutions that are intensively working toward improving public health and preventing diseases through their activities and policies. EU health strategy focuses on strengthening collaboration and coordination between the member countries, analyzing the factors affecting public health, and strengthening prevention processes, cross-border healthcare, and many other strategic areas. The availability of various financial mechanisms in the healthcare field should support access to healthcare provision, as well as increasing its availability, safety, quality, and efficiency. Health policy managers at regional and national levels are increasingly exposed to demanding decision-making processes, which prompts the construction of effective decision-making tools. Fuzzy approaches have been effectively applied in decision-making mechanisms in various sectors and can even solve difficult decision-making tasks. This study focused on constructing a tool based on a fuzzy approach, enabling effective decision-making support for the process of selection of projects in the healthcare field; this may involve many criteria regarding causal factors in health and its determinants, as well as the specifics of regional health systems.

The primary objective of our research was to develop a fuzzy decision-making support model for the evaluation and selection of healthcare projects in order to reduce the development gap between regions. The following results were obtained:

- An information model of criteria for evaluating investment projects or scientific developments in the healthcare field was developed for the first time. The set of criteria is open, and the model is not dependent on the use of a specific number of criteria. Project analysts can always add their own metrics when customizing the evaluation model for specific projects.
- A model for assessment and derivation of the level of development of regions was presented for the first time, using the example information indicator “availability and quality of services in the field of health care”. Investors are offered the opportunity to specify a “desire for a regional coefficient”, which allows them to quickly adapt the weight placed in decision-making on the regions where future projects are to be implemented.
- For the first time, a complex fuzzy decision-making support model for the evaluation and selection of healthcare projects was developed to reduce the development gap between regions. The model is based on the opinions of experts in point-based assessments. The output of the model takes the form of a ranking of projects, as well as their output evaluations, which takes into account the region of project implementation and the policy of structured funds to reduce the development gap between regions.
- The results of the study were tested in an evaluation of five real projects in the healthcare field that have been implemented at the expense of state funds in Ukraine. Furthermore, the adequacy of the complex fuzzy model developed in this study and the information models of the criteria laid down on its basis was experimentally confirmed.

The obtained results demonstrated the applied value of the model and supported the scientific hypothesis of this study.

In future studies, software could be constructed in the form of a web platform, based on a complex fuzzy model, providing an innovative tool for selecting healthcare projects for support from structural funds, considering their policies in relation to reduction of the development gaps between different regions and countries in order to achieve economic and social cohesion.

The significance of developing and implementing these tools to support decision-making processes in healthcare systems will grow in the future. Based on the demographic aging processes occurring on a global scale, it is necessary to assume that the sustainability of health and social systems in individual countries will take on increasing importance. As the population continues to age, a larger proportion of individuals will live to an older age, leading to an increase in patients with numerous comorbidities and a growing demand for health and social care services. This issue is also strongly related to limited resources. This will create pressure to optimize health systems and systematically search for opportunities to ensure the availability and provision of adequate healthcare services. Resources from EU funds will represent a strong tool for support of individual countries in meeting these increasingly demanding health goals. The use of suitable decision-making processes in the selection of optimal projects will support the effective use of resources provided by EU funds and the fulfillment of global goals in the healthcare field, thereby reducing inequalities among various countries.

The findings of this study will be highly beneficial to health policymakers; they will also aid in the formulation of strategies in the healthcare field and regional development plans, thereby supporting interdepartmental collaboration, which is necessary for effective decision-making mechanisms. The study and its results strongly advocate for the construction of national and international data platforms to develop benchmarking indicators. These indicators will play a decisive role in evaluation processes and in quantifying the effects arising from the support of activities funded by the EU.

## Data availability statement

The original contributions presented in the study are included in the article/supplementary material, further inquiries can be directed to the corresponding author.

## Author contributions

BG, MK, and VP: conceptualization and writing—original draft preparation. VP and VS: methodology and formal analysis. VP and BG: software, visualization, and project administration. TM and MK: validation. BG, MK, and TM: investigation. MK and BG: data curation. BG, MK, VP, and VS: writing—review and editing. BG: supervision. TM: funding acquisition. All authors contributed to the article and approved the submitted version.

## Footnotes

^1^Global Burden of Disease 2019. https://www.thelancet.com/gbd.

## References

[1] Clark J Horton R. A coming of age for gender in global health. Lancet. (2019) 393:2367–9. 10.1016/S0140-6736(19)30986-9 31155274

[2] Petrovic D de Mestral C Bochud M Bartley M Kivimäki M Vineis P. The contribution of health behaviors to socioeconomic inequalities in health: a systematic review. Prev Med. (2018) 113:15–31. 10.1016/j.ypmed.2018.05.003 29752959

[3] Mackenbach JP Stirbu I Roskam AJR Schaap MM Menvielle G Leinsalu M. Socioeconomic inequalities in health in 22 European countries. N Engl J Med. (2008) 358:2468–81. 10.1056/NEJMsa0707519 18525043

[4] Helgesen MK Fosse E Hagen S. Capacity to reduce inequities in health in Norwegian municipalities. Scand J Public Health. (2017) 45(18_suppl.):77–82. 10.1177/1403494817709412 28850013

[5] Luy M Minagawa Y. Gender gaps-Life expectancy and proportion of life in poor health. Health reports. (2014) 25:12. 10.1093/eurpub/cku211 25517936

[6] Williams DR. The health of men: structured inequalities and opportunities. Am J Public Health. (2008) 98(Suppl._1):S150–7. 10.2105/AJPH.98.Supplement_1.S150 18687602 PMC2518607

[7] Storm I den Hertog F Van Oers H Schuit AJ. How to improve collaboration between the public health sector and other policy sectors to reduce health inequalities?–A study in sixteen municipalities in the Netherlands. Int J Equity Health. (2016) 15:1–14. 10.1186/s12939-016-0384-y 27334297 PMC4918104

[8] Barsanti S Salmi LR Bourgueil Y Daponte A Pinzal E Ménival S. Strategies and governance to reduce health inequalities: evidences from a cross-European survey. Global Health Res Policy. (2017) 2:1–11. 10.1186/s41256-017-0038-7 29202086 PMC5683456

[9] Salmi LR Barsanti S Bourgueil Y Daponte A Piznal E Ménival S. Interventions addressing health inequalities in European regions: the AIR project. Health Promot Int. (2017) 32:430–41. 10.1093/heapro/dav101 26508665

[10] Morrison J Pons-Vigués M Díez E Pasarin MI Salas-Nicás S Borrell C. Perceptions and beliefs of public policymakers in a Southern European city. Int J Equity Health. (2015) 14:1–10. 10.1186/s12939-015-0143-5 25890326 PMC4343064

[11] Guldbrandsson K Bremberg S. Cross-sectoral cooperation at the ministerial level in three Nordic countries-With a focus on health inequalities. Soc Sci Med. (2020) 256:112999. 10.1016/j.socscimed.2020.112999 32504865

[12] Pons-Vigués M Diez È Morrison J Salas-Nicás S Hoffmann R Burstrom B. Social and health policies or interventions to tackle health inequalities in European cities: a scoping review. BMC Public Health. (2014) 14:1–12. 10.1186/1471-2458-14-198 24564851 PMC3938820

[13] Diez E Morrison J Pons-Vigués M Borrell C Corman D Burström B. Municipal interventions against inequalities in health: The view of their managers. Scand J Public Health. (2014) 42:476–87. 10.1177/1403494814529850 24756877

[14] Mardani A Hooker RE Ozkul S Yifan S Nilashi M Sabzi HZ. Application of decision making and fuzzy sets theory to evaluate the healthcare and medical problems: a review of three decades of research with recent developments. Expert Syst Appl. (2019) 137:202–31. 10.1016/j.eswa.2019.07.002

[15] Gavurova B Kelemen M Polishchuk V. Expert model of risk assessment for the selected components of smart city concept: From safe time to pandemics as COVID-19. Socioecon Plann Sci. (2022) 82:101253. 10.1016/j.seps.2022.101253 35125527 PMC8800126

[16] Stefko R Gavurova B Korony S. Efficiency measurement in healthcare work management using Malmquist indices. Polish J Manag Stud. (2016) 13:168–80. 10.17512/pjms.2016.13.1.16

[17] Bhatti MA Alyahya M. Role of leadership style in enhancing health workers job performance. Polish J Manag Stud. (2021) 24:55–66. 10.17512/pjms.2021.24.2.04

[18] Mackenbach JP Kunst AE. Measuring the magnitude of socio-economic inequalities in health: an overview of available measures illustrated with two examples from Europe. Soc Sci Med. (1997) 44:757–71. 10.1016/S0277-9536(96)00073-1 9080560

[19] Shpak N Sorochak O Gvozd M Horbal N Mykhalyuno Y Sroka W. Mechanism of competitiveness management in the public healthcare: a practical aspect. Administr Manag Public. (2022) 39:40–62. 10.24818/amp/2022.39-03

[20] Kočvarová L Barták M. Analysis of the media image and presentation of direct and hidden advertising of alcohol in selected Czech mass communication media. Study Protoc. Adiktol. (2022) 22:264–71. 10.35198/01-2022-004-0006

[21] Papadaki Š. The amount of excise tax and its effect on the consumption of alcohol and cigarettes in European countries. Adiktologie. (2022) 22:234–43. 10.35198/01-2022-004-0005

[22] Muneer A Abdemaqsoud SH Foad W Zidan M. Epidemiological study of suicidal ideation and suicidal behaviour among patients with substance use disorders in a rehabilitation and treatment centre for addiction in Dubai. Adiktologie. (2022) 22:172–83. 10.35198/01-2022-003-0002

[23] Cerešník M Cerešníková M. Risk behaviour of adolescents aged 10–15 in Slovakia. Relation to sex, age, and body mass index. Adiktologie. (2022) 22:162–70. 10.35198/01-2022-003-0001

[24] Štrangfeldová J Mališová D. Application of actuarial modeling to determine the rate of health insurance in solidary health care systems: a case of Slovakia. Administr Manag Public. (2021) 37:90–102. 10.24818/amp/2021.37-06

[25] Pisár P Priščáková S Špaček D Nemec J. Digitization as a tool of e-government in selected public services of the state: international comparison of Slovakia and the Czech Republic. Administr Manag Public. (2022) 39:111–32. 10.24818/amp/2022.39-07

[26] Stefko R Gavurova B Rigelsky M Ivankova V. Evaluation of selected indicators of patient satisfaction and economic indices in OECD country. Econ Sociol. (2019) 12:149–65. 10.14254/2071-789X.2019/12-4/917594476

[27] Liu H Li Y Tisdell CA Wang F. How does childcare by grandparents affect the health of children in China? Econ Sociol. (2021) 14:11–30. 10.14254/2071-789X.2021/14-4/1

[28] Riklikiene O Didenko O Ciutiene R Daunoriene A Ciarniene R. Balancing nurses' workload: a case study with nurse anaesthetists and intensive care nurses. Econ Sociol. (2020) 13:11–25. 10.14254/2071-789X.2020/13-2/1

[29] Mackenbach JP. Can we reduce health inequalities? An analysis of the English strategy (1997–2010). J Epidemiol Commun Health. (2011) 65:568–75. 10.1136/jech.2010.128280 21459930

[30] Hagen S Torp S Helgesen M Fosse E. Promoting health by addressing living conditions in Norwegian municipalities. Health Promot Int. (2017) 32:977–87. 10.1093/heapro/daw052 27402789

[31] Borrell LN Vaughan R. An AJPH supplement toward a unified research approach for minority health and health disparities. Am J Public Health. (2019) 109:S6–7. 10.2105/AJPH.2019.304963 30699024 PMC6356122

[32] Szymborski J Zatoński W. The activities of the Government Population Council aimed at limiting health care inequalities. J Health Inequal. (2017) 3:30–40. 10.5114/jhi.2017.69163

[33] Javakhishvili JD Kirtadze I Otiashvili D. ‘Ten years later' – Developing institutional mechanisms for drug demand reduction and addictology education in Georgia – A case study. Adiktologie. (2022) 22:35–46. 10.35198/01-2022-001-0005

[34] Orte C Amer J Gomila AM Pascual B. Combining the European Prevention Curriculum (EUPC) and Universal Treatment Curriculum (UTC) in a Master's Degree in Addictions. Adiktologie. (2022) 22:7–12. 10.35198/01-2022-001-0006

[35] Rolová G. Health literacy in residential addiction treatment programs: study protocol of a cross-sectional study in people with substance use disorders. Adiktologie. (2020) 20:145–50. 10.35198/01-2020-002-0009

[36] Gul M. A review of occupational health and safety risk assessment approaches based on multi-criteria decision-making methods and their fuzzy versions. Human Ecol Risk Assess. (2018) 24:1723–60. 10.1080/10807039.2018.1424531

[37] Shaygan A Testik ÖM. A fuzzy AHP-based methodology for project prioritization and selection. Soft Comput. (2019) 23:1309–19. 10.1007/s00500-017-2851-9

[38] Chatterjee K Hossain SA. Kar S. Prioritization of project proposals in portfolio management using fuzzy AHP. OPSEARCH. (2018) 55:478–501. 10.1007/s12597-018-0331-3

[39] Fouladgar MM Yazdani-Chamzini A Zavadskas EK Yakhchali SH Ghasempourabadi MH. Project portfolio selection using fuzzy AHP and VIKOR techniques. Transf Bus Econ. (2012) 11.

[40] Bolat B Çebi F Tekin Temur G Otay I. A fuzzy integrated approach for project selection. J Enterpr Inf Manag. (2014) 27:247–60. 10.1108/JEIM-12-2013-0091

[41] Rȩbiasz B Gaweł B Skalna I. Fuzzy multi-attribute evaluation of investments. Adv ICT Bus Ind Public Sector. (2015) 141–56. 10.1007/978-3-319-11328-9_936622615

[42] Enea M Piazza T. Project selection by constrained fuzzy AHP. Fuzzy Optimiz Decis Making. (2004) 3:39–62. 10.1023/B:FODM.0000013071.63614.3d

[43] Tulasi CL Rao AR. Resource allocation in project scheduling application of fuzzy AHP. In: Proc. Int. Conf. Technol. Business Manage. (2015). p. 512–21.

[44] Mahmoodzadeh S Shahrabi J Pariazar M Zaeri MS. Project selection by using fuzzy AHP and TOPSIS technique. Int J Ind Manuf Eng. (2007) 1:270–5.

[45] Mohammed HJ Al-Jubori IAM Kasim MM. Evaluating project management criteria using fuzzy analytic hierarchy Process. In: AIP Conference Proceedings. AIP Publishing LLC (2019). 040018 p.

[46] Tüysüz F Kahraman C. Project risk evaluation using a fuzzy analytic hierarchy process: an application to information technology projects. Int J Intell Syst. (2006) 21:559–84. 10.1002/int.20148

[47] Mohagheghi V Mousavi SM. A new framework for high-technology project evaluation and project portfolio selection based on Pythagorean fuzzy WASPAS, MOORA and mathematical modeling. Iran J Fuzzy Syst. (2019) 16:89–106. 10.22111/IJFS.2019.5022

[48] Oh J Yang J Lee S. Managing uncertainty to improve decision-making in NPD portfolio management with a fuzzy expert system. Exp Syst Appl. (2012) 39:9868–85. 10.1016/j.eswa.2012.02.164

[49] Jafarzadeh H Akbari P Abedin B. A methodology for project portfolio selection under criteria prioritisation, uncertainty and projects interdependency–combination of fuzzy QFD and DEA. Expert Syst Appl. (2018) 110:237–49. 10.1016/j.eswa.2018.05.028

[50] Mohanty RP Agarwal R Choudhury AK Tiwari MK. A fuzzy ANP-based approach to R&D project selection: a case study. Int J Prod Res. (2005) 43:5199–216. 10.1080/00207540500219031

[51] Bellahcene M Benamar FZ Mekidiche M. Ahp and wafgp hybrid model for information system project selection. Int J Anal Hierar Process. (2020) 12:228–253. 10.13033/ijahp.v12i2.76134374020

[52] Riddell S Wallace WA. The use of fuzzy logic and expert judgment in the R&D project portfolio selection process. Int J Technol Manag. (2011) 53:238–56. 10.1504/IJTM.2011.038592

[53] Mohagheghi V Mousavi SM Antuchevičiene J Mojtahed M. Project portfolio selection problems: a review of models, uncertainty approaches, solution techniques, and case studies. Technol Econ Dev Econ. (2019) 25:1380–412. 10.3846/tede.2019.11410

[54] Wu LH Wu L Shi J Chou Y-T. Project portfolio selection considering uncertainty: stochastic dominance-based fuzzy ranking. Int J Fuzzy Syst. (2021) 23:2048–66. 10.1007/s40815-021-01069-y

[55] Mardani A Jusoh A Zavadskas EK. Fuzzy multiple criteria decision-making techniques and applications–Two decades review from 1994 to 2014. Expert Syst Appl. (2015) 42:4126–48. 10.1016/j.eswa.2015.01.003

[56] Mardani A Saraji MK Mishra AR Rani P. A novel extended approach under hesitant fuzzy sets to design a framework for assessing the key challenges of digital health interventions adoption during the COVID-19 outbreak. Appl Soft Comput. (2020) 96:106613. 10.1016/j.asoc.2020.106613 32834799 PMC7410836

[57] Zare H Tavana M Mardani A Masoudian S Kamali Saraji M. A hybrid data envelopment analysis and game theory model for performance measurement in healthcare. Health Care Manag Sci. (2019) 22:475–88. 10.1007/s10729-018-9456-4 30225622

[58] Grondys K Slusarczyk O Androniceanu A. Risk assessment of the sme sector operations during the COVID-19 pandemic. Int J Environ Res Public Health. (2021) 18:4183. 10.3390/ijerph18084183 33920916 PMC8071260

[59] Androniceanu A Sabie OM Pegulescu A. An integrated approach of the human resources motivation and the quality of health services. Theor Empir Res Urban Manag. (2020) 15:42–53.

[60] Gavurova B Kubák M. The importance of evaluating inpatients' satisfaction with emphasis on the aspect of confidence. Oecon Copern. (2021) 12:821–48. 10.24136/oc.2021.027

[61] Jakubowska D Radzymińska M. Health and environmental attitudes and values in food choices: a comparative study for Poland and Czech Republic. Oecon Copern. (2019) 10:433–52. 10.24136/oc.2019.021

[62] Liu N Xu Z Skare M. The research on COVID-19 and economy from 2019 to 2020: Analysis from the perspective of bibliometrics. Oecon Copern. (2021) 12:217–68. 10.24136/oc.2021.009

[63] Bekken W Dahl E Van Der Wel K. Tackling health inequality at the local level: Some critical reflections on the future of Norwegian policies. Scand J Public Health. (2027) 45(18_suppl):56–61. 10.1177/1403494817701012 28850009

[64] Kelemen M Gavurova B Polishchuk V. A complex hybrid model for evaluating projects to improve the sustainability and health of regions and cities. Int J Environ Res Public Health. (2022) 19:8217. 10.3390/ijerph19138217 35805874 PMC9266488

[65] Simionescu M Strielkowski W Gavurova B. Could quality of governance influence pollution? Evidence from the revised Environmental Kuznets Curve in Central and Eastern European countries. Energy Rep. (2022) 8:809–19. 10.1016/j.egyr.2021.12.031

[66] Androniceanu A. The social sustainability of smart cities: urban technological innovation, big data management, and the cognitive internet of things. Geopolit Hist Int Relat. (2019) 11:110–5. 10.22381/GHIR112201910

[67] Saja P Wozny A Bednarova L. Risk management in the context of COVID-19 pandemic in an enterprise–Ishikawa cause-and-effect diagram. Syst Safety. (2021) 3:253–9. 10.2478/czoto-2021-0026

